# *Zingiber officinale* Mitigates Brain Damage and Improves Memory Impairment in Focal Cerebral Ischemic Rat

**DOI:** 10.1155/2011/429505

**Published:** 2010-12-20

**Authors:** Jintanaporn Wattanathorn, Jinatta Jittiwat, Terdthai Tongun, Supaporn Muchimapura, Kornkanok Ingkaninan

**Affiliations:** ^1^Department of Neuroscience Program, Faculty of Medicine, Khon Kaen University, Khon Kaen 40002, Thailand; ^2^Department of Pharmaceutical Chemistry and Pharmacognosy, Faculty of Pharmaceutical Sciences, Naresuan University, Phitsanulok 65000, Thailand

## Abstract

Cerebral ischemia is known to produce brain damage and related behavioral deficits including memory. Recently, accumulating lines of evidence showed that dietary enrichment with nutritional antioxidants could reduce brain damage and improve cognitive function. In this study, possible protective effect of *Zingiber officinale*, a medicinal plant reputed for neuroprotective effect against oxidative stress-related brain damage, on brain damage and memory deficit induced by focal cerebral ischemia was elucidated. Male adult Wistar rats were administrated an alcoholic extract of ginger rhizome orally 14 days before and 21 days after the permanent occlusion of right middle cerebral artery (MCAO). Cognitive function assessment was performed at 7, 14, and 21 days after MCAO using the Morris water maze test. The brain infarct volume and density of neurons in hippocampus were also determined. Furthermore, the level of malondialdehyde (MDA), superoxide dismutase (SOD), catalase (CAT), and glutathione peroxidase (GSH-Px) in cerebral cortex, striatum, and hippocampus was also quantified at the end of experiment. The results showed that cognitive function and neurons density in hippocampus of rats receiving ginger rhizome extract were improved while the brain infarct volume was decreased. The cognitive enhancing effect and neuroprotective effect occurred partly via the antioxidant activity of the extract. In conclusion, our study demonstrated the beneficial effect of ginger rhizome to protect against focal cerebral ischemia.

## 1. Introduction

Cerebral ischemia is known to produce brain damage and related behavioral deficits including memory deficit and motor disorder. It has been reported that the middle cerebral artery occlusion occurred in 10–15% of stroke patients [1]. The main areas affected by middle artery occlusion are the cerebral cortex, the hippocampus, and the striatum [2]. Memory and motor deficits are associated with interruption of blood flow to the above areas [3–7].

Free radicals are produced in cells by cellular metabolism and by exogenous agents. As age advances, production of free radicals also increases. These react with biomolecules in the brain to produce neurodegeneration and memory impairment [8]. Accumulating lines of evidence show that dietary enrichment with nutritional antioxidants could improve brain damage and cognitive function [9, 10]. Substances possessing antioxidant properties improve the cognitive function not only in normal subjects but also in cognitive deficits after stroke [11–13].

*Zingiber officinale *Roscoe or ginger, member of the family of Zingiberaceae, is widely used as a spice. Moreover, it is used in Asian traditional medicine for various purposes including stomach ache [14], nausea and diarrhea, and joint and muscle pain [15]. In addition to the effects mentioned above, ginger extract also possesses antioxidant activity [16–20], neuroprotective effect [21], and anxiolytic effect [22]. Because *Z. officinale *could scavenge free radicals, an important factor in producing brain damage induced by cerebral ischemia, we hypothesized that the *Z. officinale *extract might be able to protect against brain damage and memory impairments induced by focal cerebral ischemia via reduction of oxidative stress. The objective of this study is to determine possible effects of *Z. officinale *extract on the cognitive deficit, brain infarct volume, histopathological changes, and oxidative stress after occlusion of the right middle cerebral artery in rats.

## 2. Materials and Methods

### 2.1. Plant Material and the Preparation of the Extract

*Zingiber officinale *rhizomes were collected from Amphoe Kathum, Phitsanulok, Thailand, authenticated and prepared as analcoholic extract by Associate Professor Kornkanok Ingkaninan, Department of Pharmaceutical Chemistry and Pharmacognosy, Faculty of Pharmaceutical Sciences, Naresuan University, Thailand. A voucher specimen was also deposited at the department mentioned above. 26 kg of dried plant rhizome powder was refluxed in 45 kg of 95% ethanol for 3 hours, and the extract was filtrated. The residue was further refluxed with 35 kg of 95% ethanol for two times, and after this process, the extract was combined and dried by a freeze dryer. The percent yield of the final product was 11.54 and contained gingerol 6.78 ± 0.13%. The alcoholic extract of *Z. officinale * was prepared as suspending agent with carboxymethyl cellulose to facilitate administration via the oral route (gavage).

### 2.2. Animals

Male Wistar rats weighing 300–350 gm (8 weeks old) were obtained from National Laboratory Animal Center, Salaya, Nakorn Pathom and were housed in group of 5 per cage in standard metal cages at 22 ± 2°C on 10 : 14 h light-dark cycle. All animals were given free access to food and water ad libitum. All efforts were made to minimize animal suffering in accordance directives for the laboratory use and care of animals, issued by the European Community (EEC directive of 1986; 86/609/EEC). 

The experimental protocols were approved by the Institutional Animal Care and Use Committee.

### 2.3. Focal Cerebral Ischemia Induction

All rats were fasted for 12 hr but were allowed free access to water before surgery. Anesthesia was induced with intraperitoneal injection of thiopental sodium at dose of 50 mg/kg body weight. Focal cerebral ischemia was induced as described by Longa et al. [23]. Briefly, the right common carotid artery and the right external carotid artery were exposed through a ventral midline neck incision and were ligated proximally. A silicone-coated nylon monofilament (4-0) suture (USS DG sutures; Tyco Healthcare group LP, Connecticut, USA) with its tip rounded by heating near a flame was inserted through an arteriectomy in the common carotid artery just below the carotid bifurcation and advanced into the internal carotid artery approximately 17 to 18 mm distal to the carotid bifurcation until a mild resistance was felt. Occlusion of the origins of the anterior cerebral artery, the middle cerebral artery, and the posterior communicating artery was thereby achieved. The wound was sutured, and the rats were returned to their cages with free access to food and water. The incision sites were infiltrated with 10% povidone-iodine solution for antiseptic postoperative care.

### 2.4. Morris Water Maze Test

The water maze consisted of a metal pool (170 cm in diameter × 58 cm tall) filled with tap water (25°C, 40 cm deep) divided into 4 quadrants. In the center of 1 quadrant was a removable escape platform below the water level and covered with a nontoxic milk powder. The pool was divided into 4 quadrants (NE, NW, SE, and SW) by two imaginary lines crossing the center of the pool. For each animal, the location of invisible platform was placed at the center of one quadrant and remained there throughout training. The rats must remember location of the platform in relation to various environmental cues. Each rat was gently placed in the water facing the wall of the pool from one of the four starting points (N, E, S, or W) along the perimeter of the pool, and the animal was allowed to swim until it found and climbed onto the platform. During the training sessions, the rat was gently placed on the platform by the experimenter when it could not reach the platform in 60 s. In either case, the rat was left on the platform for 15 s and removed from the pool. The time for the animals to climb on the hidden platform was recorded as escape latency. Retention memory was also determined on the next day. The platform was removed and the animals were placed into the water maze for 60 s. The retention of the memory or the time that the animal spent to swim around the location of the platform before it was removed was recorded.

 If the spatial memory of the rat for the trained platform location is accurate, the rat will swim to the platform location and search around the exact location. Therefore, the more accurate the spatial memory is, the greater the number of times the rat will swim across precious location of platform. In each trial, the animal was quickly dried with towel before being returned to the cage [24]. All Morris water maze tests were carried out within 30 minutes after the oral administration of the test substances.

### 2.5. Determination of Infarct Volume

All animals were killed 24 hours after middle cerebral artery occlusion, and the brain was removed and sectioned at 2-mm thickness. Sections were immersed in 2% TTC (2, 3, 5-triphenyltetrazolium chloride) for 30 minutes at 37°C. Images of stained sections were digitized, and infarction volumes were determined using an indirect method.

### 2.6. Histological Procedure

Following anesthesia with sodium pentobarbital (60 mg/kg body weight), the rats were transcardially perfused with fixative containing 4% paraformaldehyde in 0.1 M phosphate buffer pH 7.3. The brains were removed and stored over night in the same fixative. They were infiltrated with 30% sucrose solution and kept at 4°C. The specimens were frozen rapidly and 30 *μ*M thick sections were cut on a cryostat. The sections were rinsed in phosphate buffer and picked up on slides coated with 0.01% of poly L-lysine.

### 2.7. Cresyl Violet Staining

Coronal sections of the brains were stained with 0.75% cresyl violet, dehydrated through graded alcohols (70, 95, 100% 2x) and xylene, and coverslipped using DPX mountant.

### 2.8. Morphological Analysis

Five coronal sections of each rat in each group were studied quantitatively. Neuronal counts in hippocampus were performed by eye using a 40X objective with final field 255 *μ*m^2^ according to the following stereotaxic coordinates: AP-4.8 mm, lateral ±2.4–6 mm, and depth 3–8 mm. The observer was blind to the treatment at the time of analysis. Viable stained neurons were identified on the basis of a stained soma with at least two visible processes. Counts were made in five adjacent fields and the mean number extrapolated to give the total number of neurons per 255 *μ*m^2^. All data were represented as number of neurons per 255 *μ*m^2^.

### 2.9. Determination of Antioxidant Enzymes Activities

In order to determine the activities of antioxidant enzymes including superoxide dismutase (SOD), catalase (CAT), and glutathione peroxidase (GSH-Px), the ischemic brains tissues were weighed and homogenized with a buffer consisting of 10 mM sucrose, 10 mM Tris–HCl, and 0.1 mM EDTA (pH 7.4) and then centrifuged at 3000 g for 15 min at 4°C. The supernatant was used for bioassays. The activity of SOD was determined using a xanthine/xanthine oxidase system for production of superoxide radical and subsequent measurement of cytochrome *c* as a scavenger of the radicals. Optical density was determined using a spectrometer (UV-1601, Shimadzu) at 550 nm. One unit of enzyme activity was defined as the quantity of SOD required to inhibit the rate of reduction of cytochrome *c* by 50% [25]. SOD activity is presented as units per milligram of protein (U mg^−1^ protein). Catalase activity in the supernatant was measured by recording the rate of decrease in H_2_O_2_ absorbance at 240 nm [26]. The activity of catalase was expressed as *μ*mol H_2_O_2_/min/mg protein. GSH-Px was determined using *t*-butylhydroperoxide as a substrate. The optical density was spectrophotometrically recorded at 340 nm. One unit of the enzyme was defined as micromoles (*μ*mol) of reduced nicotinamide adenine dinucleotide phosphate (NADPH) oxidized per minute [27]. The GSH-Px activity was expressed as U/mg protein.

### 2.10. Determination of Malondialdehyde Level

Brains regions of the rats including the cerebral cortex, striatum, and hippocampus were isolated and prepared as brain homogenates as mentioned above. The level of malondialdehyde in the brain homogenates was estimated by determining the accumulation of thiobarbituric acid reactive substances (TBARSs) according to the method of Ohkawa et al. [28].

### 2.11. Experimental Protocol

The animals were divided into 7 groups. Each group comprised of 6 animals. In this study, the doses of the test substances were selected based on our pilot study and earlier reports.

*Group I*. This served as the control group. The animals were orally given carboxymethylcellulose which was used as vehicle to dissolve the tested substance. 

*Group II*. Aricept (donepezil), an acetylcholinesterase enzyme activity inhibitor which was used as standard drug to improve memory, was orally given at dose of 1 mg/kg body weight and used as a positive control.

*Group III*. Vitamin C, a well-known antioxidant showing memory improvement, was orally administered at dose of 250 mg/kg body weight and served as positive control.

*Group IV*. Piracetam, a standard drug which increases cerebral blood flow, was administered to the rat orally at dose of 250 mg/kg body weight and served as a positive control.

*Group V-VII*. The animals were orally given *Z. officinale *at doses of 100, 200, and 300 mg/kg body weight.

The animals in all groups were given the test substances orally once daily at a period of 14 days before and 21 days after the occlusion of right middle cerebral artery (MCAO). Spatial memory was assessed at 7, 14, and 21 days after MCAO. The brain infarct volume was also determined 24 hr after MCAO.

In order to determine the effect of *Z. officinale *on the alteration of malondialdehyde level and the activities of antioxidant enzymes, the animals were divided as mentioned above. The *Z. officinale-*treated group which was selected for further study in this part was the *Z. officinale *at dose which produced optimum changes on learning memory and infarct volume. The brains of all rats were isolated and prepared the brain homogenate at 24 hr after MCAO. Then, the level of MDA and the activities of antioxidant enzymes in the brain homogenate were estimated.

### 2.12. Statistical Analysis

Data were presented as mean ± standard error of mean (S.E.M). The analysis was performed using one-way analysis of variance (ANOVA), followed by Dunnett's test. All statistical results were considered significant if *P*-value < .05.

## 3. Results

### 3.1. *Z. officinale* on Spatial Memory

**Figure 1:**
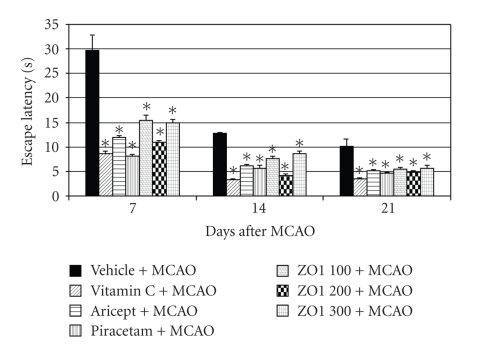
Effect of Aricept, Vitamin C, Piracetam, and various doses of ginger (*Zingiber officinale*) extract on escape latency in Morris water maze test. Values given are the mean ± S.E.M. (*n* = 6) *P-value < .05 as compared with vehicle plus MCAO.

**Figure 2:**
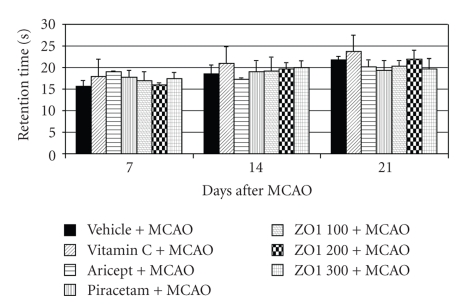
Effect of Aricept, Vitamin C, Piracetam, and various doses of ginger (*Zingiber officinale*) extract on retention time in Morris water maze test. Value given are the mean ± S.E.M. (*n* = 6).

Effect of *Z. officinale *on spatial memory was determined using escape latency and retention time as indices. It was found that all positive control groups and all doses of *Z. officinale *could significantly decrease escape latency throughout the experimental period as shown in Figure 1 (*P*-value < .05 as compared with vehicle plus MCAO). However, they failed to show the significant changes on retention time as shown in Figure 2.

### 3.2. *Z. officinale* on the Neuron Density in Hippocampus

**Figure 3:**
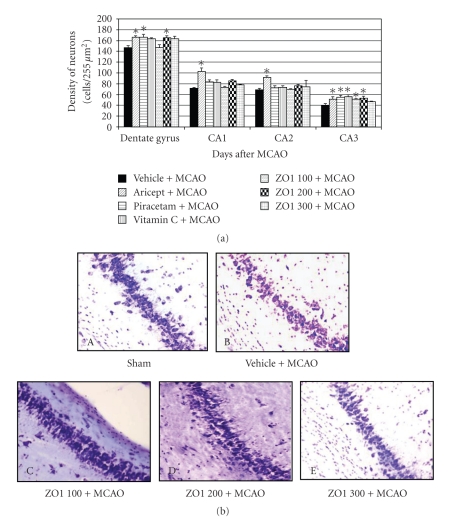
Effect of Aricept, Vitamin C, Piracetam, and various doses of ginger (*Zingiber officinale*) extract on neurons density in various subregions of hippocampus. (a) The neuron density in various areas of hippocampus (b). The photomicrographs of coronal sections of CA3 stained with cresyl violet at 40x magnification. Values given are the mean ± S.E.M. (*n* = 6) *P*-value < .05 as compared with vehicle plus MCAO.

Figure 3 shows that Aricept significantly increased the neuronal density in all subregions of the hippocampus. Piracetam also significantly increased neurons' density in the dentate gyrus and CA3 while Vitamin C significantly increased neuronal density only in CA3 (*P*-value < .05 as compared with vehicle plus MCAO). *Z. officinale *at dose of 100 mg/kg body weight could increase the neuronal density only in CA3, whereas * Z. officinale *extract at dose of 200 mg/kg body weight could increase the neuronal density both in the dentate gyrus and CA3 (*P*-value < .05 as compared with vehicle plus MCAO).

However, no significant change of this parameter was observed after *Z. officinale *administration at dose of 300 mg/kg body weight.

### 3.3. *Z. officinale* on Brain Infarct Volume

**Figure 4:**
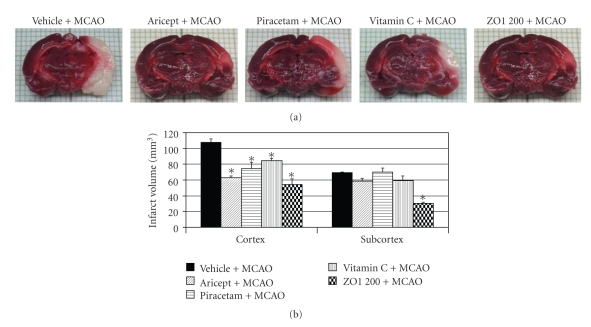
Effect of Aricept, Vitamin C, Piracetam, and ginger (*Zingiber officinale*; ZO1 200) extract at dose of 200 mg/kg body weight on brain infarct volume. Brain infarct volume was determined using TTC staining. Values given are the mean ± S.E.M. (*n* = 6) *P-value < .05 as compared with vehicle plus MCAO.

Previous study had demonstrated that the brain impairments were associated with the severity of brain infarction. Hence, we determined the brain infarct volume after *Z. officinale *administration. The results in Figure 4 show that both Aricept and Piracetam markedly decreased the infarct volume in the cortical area, but no significant change was observed in subcortical areas (*P*-value < .05 as compared with vehicle plus MCAO). No significant change was observed after Vitamin C administration. Surprisingly, *Z. officinale *at a dose of 200 mg/kg body weight (dose which produced optimum change) could reduce the brain infarct volume better than all positive control-treated groups in this study. The plant extract could decrease the brain infarct volume in both cortical and subcortical areas (*P*-value < .05 as compared with vehicle plus MCAO).

### 3.4. *Z. officinale* on the Level of Malondialdehyde and Antioxidant Enzymes

**Figure 5:**
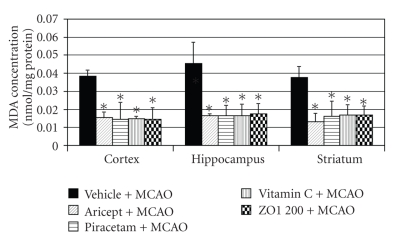
Effect of Aricept, Vitamin C, Piracetam, and ginger (*Zingiber officinale*; ZO1 200) extract at dose of 200 mg/kg body weight on the level of malondialdehyde (MDA), a product of lipid peroxidation in cerebral cortex, hippocampus, and striatum. Values given are the mean ± S.E.M. (*n* = 6) *P-value < .05 as compared with vehicle plus MCAO.

Based on the neuroprotective effect of *Z. officinale *extract, we also further investigated the effect of *Z. officinale *on the malondialdehyde, an indirect marker of the oxidative damage of various macromolecules as shown in Figure 5. It was found that all positive control groups and *Z. officinale *significantly decreased malondialdehyde level in cerebral cortex, striatum, and hippocampus (*P*-value < .05 as compared with vehicle plus MCAO).

**Figure 6:**
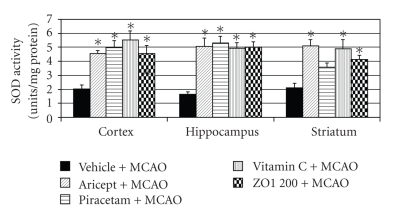
Effect of Aricept, Vitamin C, Piracetam, and ginger (*Zingiber officinale*; ZO1 200) extract at dose of 200 mg/kg body weight on the activity of superoxide dismutase (SOD) in cerebral cortex, hippocampus, and striatum. Values given are the mean ± S.E.M. (*n* = 6) *P-value < .05 as compared with vehicle plus MCAO.

**Figure 7:**
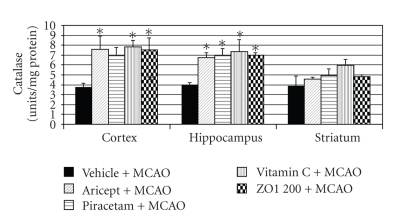
Effect of Aricept, Vitamin C, Piracetam, and ginger (*Zingiber officinale*; ZO1 200) extract at dose of 200 mg/kg body weight on the activity of catalase (CAT) in cerebral cortex, hippocampus, and striatum. Values given are the mean ± S.E.M. (*n* = 6) *P-value < .05 as compared with vehicle plus MCAO.

**Figure 8:**
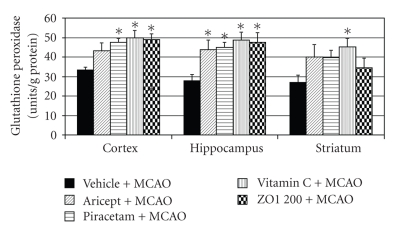
Effect of Aricept, vitamin C, Piracetam, and ginger (*Zingiber officinale*; ZO1 200) extract at dose of 200 mg/kg body weight on the activity of glutathione peroxidase (GSH-Px) in cerebral cortex, hippocampus, and striatum. Values given are the mean ± S.E.M. (*n* = 6) *P-value < .05 as compared with vehicle plus MCAO.

In addition, Figure 6 shows that Aricept and Vitamin C could increase the activity of SOD in all areas mentioned earlier while Piracetam significantly increased the activity of SOD only in the cerebral cortex and hippocampus. Surprisingly, *Z. officinale *also increased the activity of SOD in all areas mentioned above at the same magnitude as that induced by Aricept and Vitamin C. Moreover, it appeared to show better effect than Piracetam. The positive control group and *Z. officinale *also increased the activity of CAT in both cerebral cortex and hippocampus as shown in Figure 7 (*P*-value < .05 as compared with vehicle plus MCAO). In this study, we also determined the effect of *Z. officinale *on the activity of glutathione peroxidase as shown in Figure 8. Aricept and Piracetam significantly increased the activity of GSH-Px in cerebral cortex and hippocampus while Vitamin C could significantly increase the activity of this enzyme in all areas (*P*-value < .05 as compared with vehicle plus MCAO). The *Z. officinale *extract also significantly increased the activity of GSH-Px in cerebral cortex and hippocampus (*P*-value < .05 as compared with vehicle plus MCAO).

## 4. Discussion

Our results demonstrated for the first time that *Z. officinale *could protect ischemic brain damage in a rat model of focal cerebral ischemia. Moreover, it also reduced cognitive deficits induced by focal cerebral ischemia.

MCAO had been reported to induce a number of important cellular changes including increase in intracellular calcium and excessive free radicals leading to brain damage and impairments including cognitive deficit [29]. MCAO has been previously reported to induce brain damage and neuronal death in the hippocampus [30]. It was found that the neurodegeneration in hippocampus was associated with spatial memory impairment [31–33]. In addition, MCAO also decreased acetylcholine [34]. These findings were in agreement with our data. Aricept, an acetylcholineseterase inhibitor, was previously shown to improve brain damage and memory impairment in MCAO [35]. It was found that Aricept inhibited not only acetylcholinesterase activity but also oxidative stress [36]. Therefore, the neuroprotective effect of Aricept might be possibly related to its antioxidant activity. However, its effect to inhibit acetylcholinesterase activity still could not be omitted. It was previously reported that during a memory task, the cerebral blood flow increased and the disruption of cerebral blood flow resulted in memory impairment [37]. Our results also demonstrated that Piracetam, a drug reputed to increase cerebral blood flow, improved the cognitive function induced by MCAO. Therefore, the present results were consistent with previous study which reported the neuroprotective and nootropic effect of this drug and its ability to improve symptoms of acute stroke [38]. Moreover, the current study also showed that the interruption of cerebral blood flow resulted in the insufficient supply of ascorbic acid in the brain and inadequate delivery of oxygen and glucose [39, 40], and finally leading to brain damage and brain impairment. Vitamin C had been reported to be involved in almost the important neurochemical processes during cerebral ischemia [41]; therefore, supplementation of Vitamin C or ascorbic could improve brain damage and impairment as shown in this study.

Surprisingly, *Z. officinale *could increase the neurons' density in hippocampus and improved the spatial memory. Although the neurodegeneration in hippocampus is reported to be associated with the spatial memory deficit [32, 33], the present findings showed that the improvement of spatial memory was not tightly correlated with the increase of neuronal density in the hippocampus. It was found that all doses of *Z. officinale *could improve spatial memory while *Z. officinale *at dose of 300 mg/kg body weight failed to show neuroprotective effect in this area. This indicates that the cognitive enhancing effect of *Z. officinale *occurred not only due to increasing neuronal density in hippocampus but also due to other mechanisms. *Z. officinale *has previously been reported to induce vasodilation [42]. Therefore, it might be possible that *Z. officinale *could enhance cerebral blood flow resulting in the improvement of spatial memory as Piracetam. A dose which provided the most beneficial effect was 200 mg/kg body weight. The lower dose failed to show the neuroprotective effect in dentate gyrus while the *Z. officinale *at dose of 200 mg/kg body weight could increase the neuronal density in this area. One possible explanation for this phenomenon might be related to the insufficient level of *Z. officinale *to reach the therapeutic level. On the other hand, *Z. officinale *at dose of 300 mg/kg body weight also failed to show a neuroprotective effect in hippocampus. This might occur because the *Z. officinale *extract used in this study was the crude extract; therefore, increasing the dose of the extract might also increase the concentration of some ingredients which masked the effect of the active ingredient. 

Our results also demonstrated that *Z. officinale *at dose of 200 mg/kg body weight could mitigate the brain infarct volume and could decrease oxidative stress by increasing the activity of SOD in cerebral cortex, hippocampus, and striatum and increased the activities of CAT and GSH-Px in cerebral cortex and hippocampus resulting in the decrease of lipid peroxidation level in all areas mentioned earlier. Therefore, the neuroprotective effect of Z*. officinale *extract might be related to its antioxidant effect. 

In conclusion, our data suggested that *Z. officinale * possessed the protective effect against focal cerebral ischemia induced by the occlusion of right middle cerebral artery. It could attenuate the memory impairment, neurodegeneration, and brain infarct volume in this condition. The cognitive enhancing effect and neuroprotective effect of *Z. officinale *appeared to show almost the same magnitude as the positive control groups used in this study. Moreover, *Z. officinale *also showed multiple sites of action. Therefore, *Z. officinale *at the correct dose is a potential novel candidate for developing food supplement against focal cerebral ischemia. However, further clinical trial study is still required.
