# Corrigendum to “*Zingiber officinale* Mitigates Brain Damage and Improves Memory Impairment in Focal Cerebral Ischemic Rat”

**DOI:** 10.1155/2022/9761293

**Published:** 2022-05-31

**Authors:** Jintanaporn Wattanathorn, Jinatta Jittiwat, Terdthai Tongun, Supaporn Muchimapura, Kornkanok Ingkaninan

**Affiliations:** ^1^Department of Neuroscience Program, Faculty of Medicine, Khon Kaen University, Khon Kaen 40002, Thailand; ^2^Department of Pharmaceutical Chemistry and Pharmacognosy, Faculty of Pharmaceutical Sciences, Naresuan University, Phitsanulok 65000, Thailand

In the article titled “*Zingiber officinale* mitigates brain damage and improves memory impairment in focal cerebral ischemic rat” [1], there are concerns in relation to duplication in Figure 1(a), in which the panels for Aricept + MCAO and ZO1 + MCAO are the same. The authors apologized for their error and explained that this was inadvertently introduced during the preparation of the figures and that the Aricept panel was the original image.

The authors provided the replicate images for each treatment group, which are available in the supplementary materials. (available here)

Figure 1 has been corrected as follows to remove the duplicated image.

## Figures and Tables

**Figure 1:**
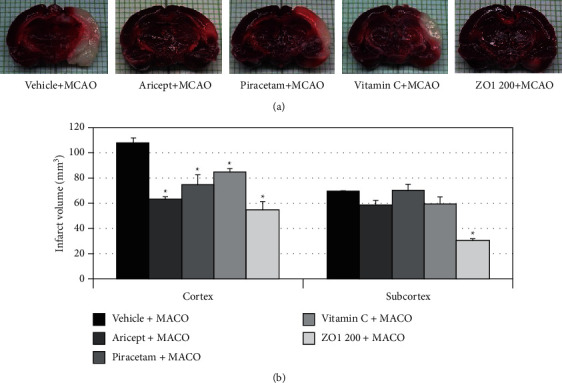
Effect of Aricept, Vitamin C, Piracetam, and ginger (*Zingiber officinale*; ZO1 200) extract at dose of 200 mg/kg body weight on brain infarct volume. (a) Representative photographs of TTC-stained brain sections in each group. (b) Effect of Aricept, Vitamin C, Piracetam, and *Zingiber officinale* (ZO1 200) extract at dose of 200 mg/kg body weight on brain infarct volume. The results are expressed as mean ± S.E.M. (*n* = 6) ^*∗*^*P*-value < .05 as compared with vehicle plus MCAO.

## Supplementary Materials

Supplementary Materials Replicate images of the effect of Aricept, Vitamin C, Piracetam, and ginger (*Zingiber officinale*; ZO1 200) extract at dose of 200 mg/kg body weight on brain infarct volume. Brain infarct volume was determined using TTC staining.

## References

[1] Wattanathorn J., Jittiwat J., Tongun T., Muchimapura S., Ingkaninan K. (2011). *Zingiber officinale* mitigates brain damage and improves memory impairment in focal cerebral ischemic rat. *Evidence-based Complementary and Alternative Medicine*.

