# Supplementary material for: Corrigendum to “Zingiber officinale Mitigates Brain Damage and Improves Memory Impairment in Focal Cerebral Ischemic Rat”
Source: Evid Based Complement Alternat Med. 2022 May 31;2022:9761293. doi: 10.1155/2022/9761293 (PMC9173990; doi:10.1155/2022/9761293)
Supplement: Supplementary Materials — Replicate images of the effect of Aricept, Vitamin C, Piracetam, and ginger (Zingiber officinale; ZO1 200) extract at dose of 200 mg/kg body weight on brain infarct volume. Brain infarct volume was determined using TTC staining. [file 9761293.f1.docx]

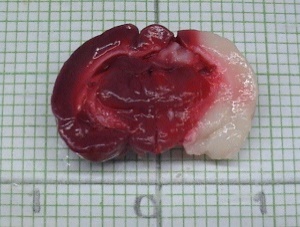

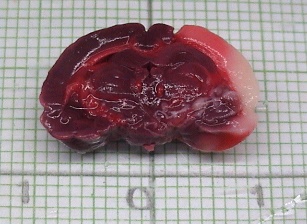

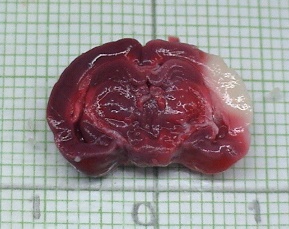


**Vehicle+MCAO**

**Aricept+MCAO**

**Piracetam+MCAO**

**Vitamin C+MCAO**

**ZO1 200+MCAO**


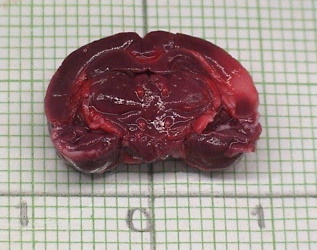

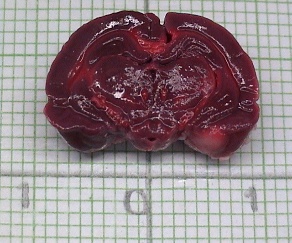


(a)


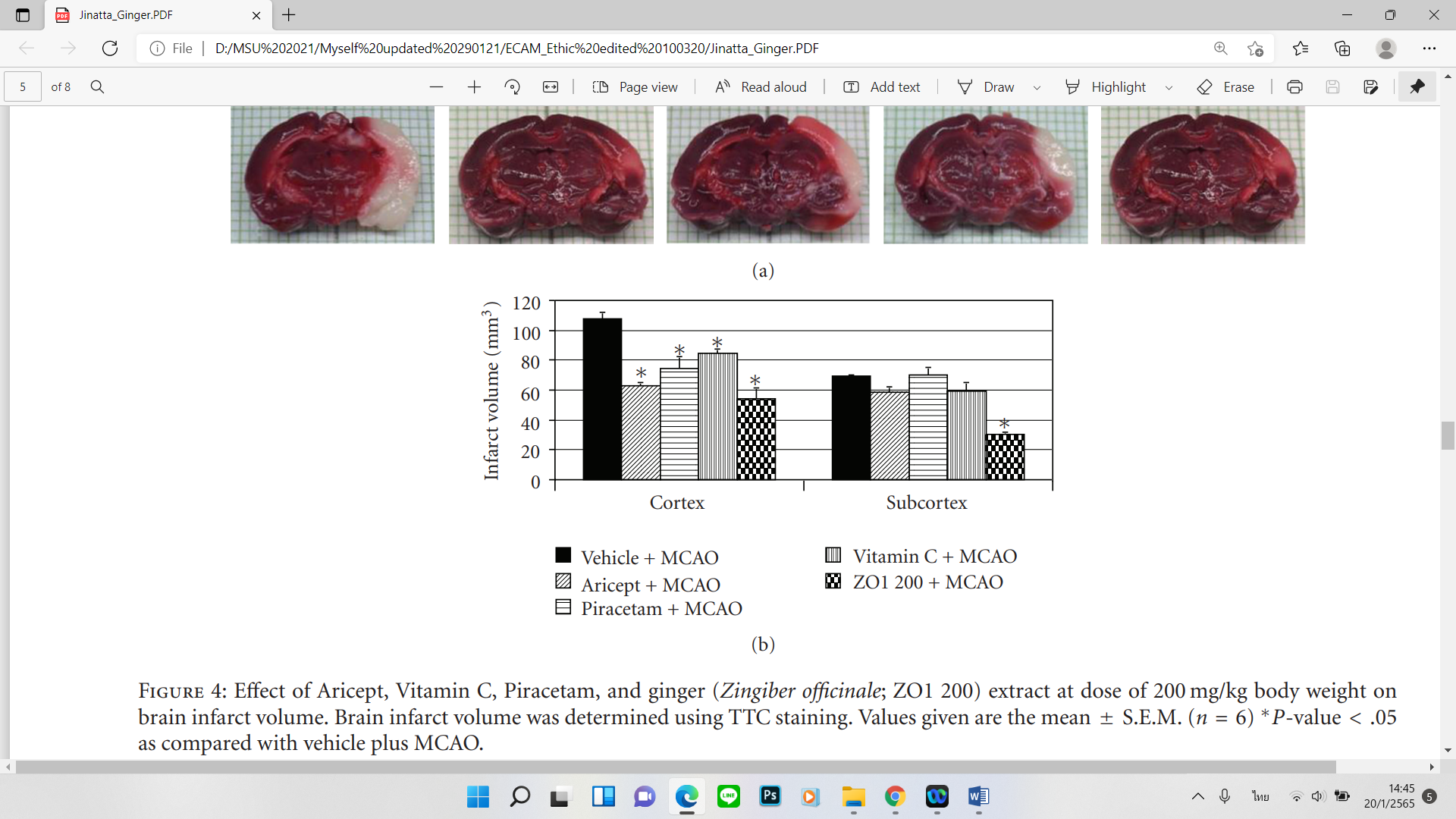


(b)

Figure 4: Effect of Aricept, Vitamin C, Piracetam, and ginger (*Zingiber officinale*; ZO1 200) extract at dose of 200 mg/kg body weight on brain infarct volume. (a) Representative photographs of TTC-stained brain sections in each group. (b) Effect of Aricept, Vitamin C, Piracetam, and *Zingiber officinale* (ZO1 200) extract at dose of 200 mg/kg body weight on brain infarct volume. The results are expressed as mean ± S.E.M. (n = 6) ^∗^*P*-value < .05 as compared with vehicle plus MCAO.
